# Analysis of respirable dust exposure data collected at a Zambian copper mine between 2017 and 2022

**DOI:** 10.3389/fpubh.2023.1288485

**Published:** 2024-01-31

**Authors:** Mwaba Sifanu, Kennedy K. Kalebaila, Patrick Hayumbu, Lubinda Nabiwa, Stephanus J. L. Linde

**Affiliations:** ^1^School of Mathematics and Natural Sciences, Copperbelt University, Kitwe, Zambia; ^2^Occupational Hygiene and Health Research Initiative, North-West University, Potchefstroom, South Africa

**Keywords:** dust exposure, mining shafts, monitoring program, concentrator plant, Zambian copper mining, retrospective data

## Abstract

Mine workers are occupationally exposed to respirable dust, which can cause irreversible lung diseases and controlling exposure concentrations to as low as reasonably practicable is, therefore, essential. To implement exposure reduction strategies and adequately manage exposure to hazardous chemicals, exposure needs to be measured and recorded according to a standard exposure management plan. This study aimed to assess the available respirable dust exposure data measured between 2017 and 2022 in various work areas and job categories at two mining shafts and a concentrator plant of a Zambian copper mine. Additionally, the monitoring program implemented at the mine was assessed for appropriateness. Descriptive data analysis was used to create an exposure matrix comprising 253 exposure measurements. Approximately 5.6% of the measured concentrations exceeded the South African time-weighted average occupational exposure limit (TWA-OEL) of 3 mg/m^3^. The geometric means of respirable dust exposure for shaft A, shaft B and the concentrator plant were 0.44 mg/m^3^, 0.44 mg/m^3^, and 0.68 mg/m^3^, respectively. The work areas with the highest maximum exposure results were the tipping area (18.0 mg/m^3^) at shaft A, the sump and waste bin (8.05 mg/m^3^) at shaft B and the screening (10.8 mg/m^3^), secondary crusher (14.0 mg/m^3^), foreign ore bin (4.43 mg/m^3^), and tertiary crusher (5.07 mg/m^3^) areas at the concentrator plant. It was found that the monitoring strategy implemented at the mine was flawed and did not collect a sufficient number of samples in each area during each year to make rigorous statistical assessment possible. This study highlights the sampling strategy’s shortcomings and recommends adopting a standardized monitoring strategy, such as EN689, to improve the respirable dust monitoring program at the mine. Additionally, this strategy can be implemented across Zambia and, if implemented correctly, it has the potential to be improve exposure monitoring across the country as no standard sampling strategy is currently enforced by the Government.

## 1 Introduction

Particulate matter is the term used for airborne particles that can contain both organic and inorganic materials, including smoke, dust, pollen dust, and liquid droplets (1), while dust is the general term used to describe solid materials with different sizes, shapes and densities (2). Occupational exposure to dust occurs in many different operations, such as mining, agriculture, construction, quarry, brick and tile industries (3). Airborne dust is generated during rock movement, breaking, crushing and shaping. Whether respirable dust exposure is short or long-term, it can cause respiratory diseases ranging from acute to chronic (4, 5). Respirable dust may contain substances like silica, likely to cause silicosis and pneumoconiosis. At the same time, larger-sized fractions (inhalable and thoracic) are likely to cause bronchitis and chronic obstructive pulmonary diseases (6). In occupational environments, dust particles are bundled into three different fractions, and the aerodynamic diameter of an inhaled dust particle determines where it will deposit in the respiratory tract, these include, inhalable, thoracic and respirable (2). Inhalable dust ranges between 1 and 100 μm and is the fraction that penetrates past the mouth and nose. The thoracic fraction is the mass fraction that penetrates beyond the larynx (50% penetration at 10 μm), and the respirable fraction is the mass fraction that penetrates the unciliated airways (50% penetration at 4 μm) (1, 6).

The mining industry exposes workers in development, production and processing sections to various occupational hazards, including respirable dust (7). There is no 8-h time-weighted average (TWA) occupational exposure limit (OEL) for respirable dust in Zambia. Therefore, this study adopted the TWA-OEL of 3 mg/m^3^ used by the South African Mine Health and Safety Act (No. 29 of the 1996) (8, 9). This limit is the same as the American Conference of Governmental Industrial Hygienists (ACGIH) health-based threshold limit value (TLV) (8–10). A systematic sampling strategy is essential to help identify high exposure areas in a statistically accurate manner, the exposure concentrations above the exposure limit are considered significant because they indicate potential risks associated with specific jobs. They are also crucial during decision-making processes and essential to protecting workers’ health and well-being (11, 12).

A number of studies indicating that respirable dust exposure can cause occupational respiratory diseases have been conducted and they highlight specific jobs in the mining industry where workers may be highly exposed to respirable dust and respirable crystalline silica dust (13–15). All mine workers are potentially at risk of exposure to respirable dust in surface or underground operations. Mining crews, team leaders, tip operators, drill operators and locomotive drivers are considered to be significant dust exposure jobs (16, 17). A retrospective study conducted by Dahmann et al. (13) provided information on miners’ exposure to inhalable dust, respirable dust, crystalline silica and heavy metals between 1990 and 2002. The study revealed all underground jobs had high respirable dust and respirable crystalline silica exposure, reaching TWAs of 20 mg/m^3^ and 2 mg/m^3^, respectively.

Similarly, a study by Hayumbu et al. (14) measured respirable dust and respirable crystalline silica concentrations at two Zambian copper mines. The study reported mean concentrations for respirable dust 0.99 mg/m^3^ (range 0–7.67 mg/m^3^) for one mine and 0.87 mg/m^3^ (range 0–6.94 mg/m^3^) for another mine. The mean respirable crystalline silica concentrations were 0.14 mg/m^3^ and 0.06 mg/m^3^, and ranged between 0 and 1.30 mg/m^3^ and 0–0.317 mg/m^3^, respectively. Hayumbu et al. (14) further concluded that the mining companies had substandard dust monitoring programs, which may increase miners’ risk of non-malignant disease. A retrospective analysis conducted in Australia by Rumchev et al. (18) investigated the association between exposure to inhalable and respirable dust and respiratory health problems among mine workers between 2001 and 2012. The study found a decline in exposure to inhalable and respirable dust over the 12 years despite workers exhibiting high prevalence of phlegm and cough. Similarly, a study by Mannetje et al. (19) reported a decline in dust exposure over time for a pooled analysis of studies conducted from different parts of the world. In the construction industry, Mastrantonio et al. (15) assessed inhalable, respirable, and respirable crystalline silica dust exposure among construction workers (bricklayers, scaffolders and carpenters) in rebuilding activities. The results revealed that geometric means for inhalable dust, respirable dust and respirable crystalline silica were 4.3 mg/m^3^, 0.25 mg/m^3^ and 0.004 mg/m^3^, respectively, which implied that all jobs were below the ACGIH TLV of 3 mg/m^3^ for dust and 0.025 mg/m^3^ for crystalline silica. Furthermore, Zilaout et al. (20) assessed trends of respirable dust and respirable crystalline silica concentrations within the European industrial mining sector over a 15-year period. The study collected approximately 32,000 personal exposure measurements during the 29 sampling campaigns. The results revealed an overall statistically significant downward trend of −9.0% and − 3.9% for respirable dust and crystalline silica, respectively. However, when analyses were stratified by time, no downward trends were observed between 2008 and 2012 which the authors attributed to the global economic crisis.

The analysis of respirable dust exposure needs to follow a standardized protocol to ensure its validity and to promote confidence in the generated results. One such a standard is EN689:2018 (Workplace exposure measurement of exposure by inhalation to chemical agents: strategy for testing compliance with occupational exposure limit values). This standard is applicable for measuring procedures that fulfill the requirements of the EN 482:2021 (Workplace exposure procedures for determination of concentration of chemical agents - Basic performance requirements) (21). The EN689 standard specifies four steps which include step (1) basic characterization involving identification of chemical agents and other information relation to safety such as chemical composition, review of workplace factors and estimation of similar exposure groups, step (2) the constitution of exposure groups and specifying the measuring procedures, step (3) performing exposure measurements and step (4) the validation of results (22). The representative measurement of occupational exposure to chemicals is difficult due to many factors that may cause variability in results (22). Therefore, it is important to collect exposure measurements according to a validated strategy that will lead to exposure information that can be trusted and that can be used to make accurate decisions in the exposure management process. Though not standardized a dust monitoring program has been implemented at the mining site specified in this study. Exposure measurements are taken annually, but not much is being done with the data in terms of statistical analysis. Therefore, the ultimate aim of this study was to analyze the available data on respirable dust exposure reported between 2017 and 2022 at two mining shafts and a concentrator plant of a Zambian copper mine. This was done according to work areas and job categories where respirable dust exposure was reported to occur. Additionally, the sampling strategy used by the mine was also evaluated to determine its appropriateness. Because it is the first study of its kind in the mining sector in Zambia, other mining companies will be able to use this study’s data for comparisons in the future.

## 2 Methods

The work areas included in the current study comprised of both underground and surface operations located in the Copperbelt Province of Zambia. The Copperbelt Province is characterized by a river basin rich in minerals, a major valley and high plateau, therefore there are a lot of mining activities that that place in this province. Until 2017, no systematic determination of dust concentrations had been performed at the mining site. Only occasional measurements for total dust were performed, which did not allow significant evaluation of dust exposures at the mine. From 2017 onwards, personal respirable dust exposure measurements were collected from different workers in various jobs and the data for this study was obtained from the mine’s ongoing surveillance monitoring program. Between 2017 and 2022, a total of 253 homogeneous personal respirable dust exposure samples were collected with no particular focus on the sampling areas, and all areas at the two mining shafts and the concentrator plant were included (see Table 1).

**Table 1:** Summary of respirable dust exposure concentration for work areas between 2017 and 2022.

Area	Number of samples per year						
	Work areas	2017	2018	2019	2020	2021	2022
Mining shaft A	Ore tip	0	1	2	5	8	3
	Primary crusher	0	0	0	3	1	1
	Tipping	0	3	4	20	9	4
	Tramming	0	3	2	8	8	1
	Loading box	0	0	0	4	3	2
	Sump and waste bin	0	0	0	0	0	0
	Pump and filter chamber	0	0	0	0	0	0
	Total	0	4	8	40	29	17
Concentrator	Tertiary crusher	1	3	3	7	5	0
	Dams	0	0	4	0	0	0
	Flotation	0	0	0	2	1	3
	Foreign ore bin	2	0	3	4	0	0
	Screening	1	1	4	0	1	0
	Secondary crusher	1	0	7	0	1	0
	Total	5	4	21	13	8	3
Mining shaft B	Ore tip	0	2	23	15	5	0
	Primary crusher	0	0	0	0	0	0
	Tipping	0	0	0	0	0	0
	Tramming	0	0	1	0	0	1
	Loading box	0	0	3	2	0	0
	Sump and waste bin	0	3	10	15	7	4
	Pump and filter chamber	0	0	0	10	3	0
	Total	0	5	29	40	10	5

### 2.1 The mining shafts and concentrator plant work areas

Copper-containing ore from the earth is extracted at the two underground mining shafts (shaft A and B). Mining is performed by blasting the rock face, after which the ore is trammed (transported) by loaders or locomotives to the tipping sections, further broken down into smaller pieces. The broken-down pieces are thereafter taken to the primary crushers, where large pieces of copper ore are broken down into smaller pieces via the loading box. Following the primary crusher, the copper ore is sent to the sumps and waste bins before it is taken to the surface bins, and it is from there that the train is used to transport it to the foreign ore bin (FOB) at the concentrator. After that, it goes to the screening section, where the crushed ore is screened for waste material like wires and scrap metal, which are manually removed as it passes via the conveyor belts to the secondary crusher. Further crushing occurs at the secondary crusher before the copper ore is taken to the tertiary crusher for milling. After milling, the copper ore is taken to the flotation section, where other minerals like nickel are separated from copper and waste is taken to the dams.

### 2.2 Job titles at the mining shafts and concentrator plant

The job titles found at the mining shafts and concentrator plant were as follows: Belt attendants ensure that conveyor belts are fully functional as ore is transported from one section to another. Loader and locomotive operators are underground drivers responsible for driving dump trucks and locomotives after loading ore. Operators are found to operate different machinery at the mining site, i.e., crushers and tipping. The person in charge ensures that all places of work in a section are safe before and during a shift. Using machinery, rock breakers reduce large rocks of copper ore into smaller manageable pieces. Shift bosses ensure the smooth running of the shift in all sections of the entire shaft. The section boss reports to the shift boss on how the shift and operations in a given section are progressing and ensures all workers adhere to safety standards. The skip man is responsible for loading ore in the loading box and ensuring it is safely sent to the crusher section. The whistle man works hand in hand with the locomotive driver to signal the driver of any dangers which may be present. A workman is a helper who mainly performs manual work at the mine, i.e., clearing haulages for mobile equipment. The shunter transports copper ore at the surface from the shafts to the concentrator using a surface locomotive (train). To identify similar exposure groups (SEGs), the study used the mines baseline risk assessment. This has been summarized in Supplementary Table S1.

### 2.3 Exposure data

Anonymized personal respirable dust exposure data measured from 2017 to 2022 at the mining shafts and the concentrator plant were obtained from the mine site data system. The 8 h time weighted average exposure data were available for the mining shafts from 2018 to 2022 and for the concentrator plant from 2017 to 2022. The exposure was measured using the National Institute for Occupational Safety and Health (NIOSH) gravimetry method (0600) (23). The personal measurements were collected using a 37 mm polyvinyl chloride (PVC) membrane filter (0.5 μm pore size), two-part cassette, 10 mm conductive nylon cyclone (Dorr-Oliver Style) with holder and a personal air sampling pump (Gill Air Plus) at a flow rate of 1.7 L/min with the. Before and after sampling, the air flow rate was calibrated using a Gilibrator-2 air flow calibrator (Sensidyne, St. Petersburg, Florida, USA). A total of 253 measurements were available for use in the study. The limit of detection (LOD) and limit of quantification (LOQ) for the method was 0.01 mg/m^3^ and 0.012 mg/m^3^, respectively.

### 2.4 Statistical analysis

An exposure matrix was created for the dataset by grouping the data from the concentrator into work areas and the two mining shafts according to similar work areas. The mining shafts were grouped into seven work areas, i.e., ore tip, primary crusher, tramming, tipping, loading box, sump and waste bin, and pump and filter chamber. The work areas for the concentrator plant were partitioned into six work areas, i.e., tertiary crusher, dams, flotation, foreign ore bin (FOB), screening and secondary crusher.

To provide a preliminary description of the dataset, minimum and maximum exposure concentrations, arithmetic mean (AM), standard deviation (SD), geometric mean (GM), geometric standard deviation (GSD) and 95% upper confidence limits of the GM were calculated for each category. The data was log-normal and was log-transformed before conducting a one-way analysis of variance (ANOVA) to determine significant differences between the work areas and the job titles. All the figures and computations were generated using GraphPad Prism® version 5.03 (GraphPad Software).

## 3 Results

Approximately 79% of the personal respirable dust exposure measurements collected between 2017 and 2022 were obtained from the two mining shafts, and 21% was collected at the concentrator.

### 3.1 Personal exposure in the mining shafts and concentrator plant

A summary of personal respirable dust exposure data for the two mining shafts and the concentrator plant is presented in an exposure matrix (see Table 2). The range of respirable dust exposure at the mine was between 0.03 mg/m^3^ and 18.0 mg/m^3^ (GM = 0.44 mg/m^3^) for mining shaft A, between 0.02 mg/m^3^ and 8.05 mg/m^3^ (GM = 0.44 mg/m^3^) for mining shaft B, and between 0.05 mg/m^3^ and 14.0 mg/m^3^ (GM = 0.68 mg/m^3^) for the concentrator plant. The number of exposure concentrations above the TWA-OEL for the mine was 14 (five from mining shaft A, two from mining shaft B and seven from the concentrator plant). The personal respirable dust concentrations from the various work areas are also summarized in Table 2. The percentage of respirable dust exposure measurements that exceeded the TWA-OEL at the mining shafts and the concentrator plant between 2017 and 2022 was 5.6% (2.0% mining shaft A, 0.8% mining shaft B and 2.8% concentrator plant) and six areas within the mining shafts and four areas from the concentrator reported measurements that exceeded the TWA-OEL. Concerning jobs that reported measurements greater than the TWA-OEL, four job categories (person in charge, rock breaker, skip man, workman) at the shafts and five job categories (crusher belt attendant, operator, shift boss, shunter, workman) at the concentrator plant reported measurements above the TWA-OEL.

**Table 2:** Summary of personal respirable dust concentrations (TWA mg/m^3^) per mining shaft, per work area and job title between 2017 and 2022.

Mining shaft	work area	Job title	*n*	Min	Max	AM	SD	GM	GSD	95% UCI	% n* > OEL
Mine (total)			253	0.02	18.0	0.92	1.83	0.43	3.21	0.49	6
Mining shaft A			95	0.03	18.0	0.88	1.94	0.44	2.97	0.55	5
Concentrator			54	0.05	14.0	1.57	2.60	0.68	3.67	0.96	13
Mining shaft B			104	0.02	8.05	0.62	0.95	0.44	2.97	0.42	2

Mining shaft A	Tipping	40	0.03	18.0	1.03	2.83	0.42	3.04	0.60	5
	Tramming	22	0.60	3.22	0.73	0.80	0.44	2.89	0.70	5
	Ore tip	19	0.05	3.39	0.93	0.88	0.55	3.39	0.97	5
	Loading box	9	0.13	3.03	0.62	0.91	0.39	2.33	0.75	11
	Primary crusher	5	0.12	2.29	0.64	0.09	0.34	3.00	1.49	0
Concentrator	Tertiary crusher	19	0.09	5.07	1.15	1.18	0.72	2.89	1.23	5
	Dams	4	0.17	0.73	0.46	0.23	0.40	1.86	1.09	0
	Flotation	6	0.08	0.49	0.41	1.15	0.19	1.99	0.39	0
	Foreign ore bin	9	0.88	4.43	4.35	1.67	0.77	4.24	2.35	22
	Screening	7	0.27	10.8	3.32	4.37	1.34	4.66	6.73	29
	Secondary crusher	9	0.22	14.0	3.26	4.67	1.54	3.64	4.54	22
Mining shaft B	Ore tip	45	0.02	3.09	0.66	0.70	0.39	3.05	0.55	2
	Loading box	5	0.20	0.38	0.28	0.75	0.28	1.31	0.38	0
	Tramming	2	0.74	0.76	0.70	0.76	0.75	1.02	0.89	0
	Sump and waste bin	39	0.03	8.05	0.70	1.34	0.31	3.53	0.46	3
	Pump and filter chamber	13	0.06	0.67	0.29	0.18	0.25	1.97	0.37	0

Mining shaft A	Tipping	Belt attendant	7	0.13	3.03	0.66	0.90	0.39	2.33	0.75	14
		Loader driver	4	0.36	0.83	0.56	0.54	0.54	1.41	0.93	0
		Loco driver	7	0.20	1.09	0.53	0.47	0.47	1.78	0.79	0
		Operator	2	0.17	2.25	1.21	0.62	0.62	6.21	82,767	0
		Person in charge	4	0.26	18.0	4.81	0.97	0.97	7.37	23.2	25
		Rock breaker	6	0.16	2.01	0.73	0.39	0.39	2.93	1.19	0
		Section boss	2	0.26	0.33	0.29	0.29	0.29	1.18	1.33	0
		Skip man	5	0.10	1.96	0.52	0.25	0.25	3.31	1.1	0
		Whistle man	3	0.21	0.61	0.61	0.42	0.42	1.82	1.84	0
	Tramming	Loader driver	4	0.28	1.24	0.82	0.40	0.719	1.92	2.03	0
		Loco driver	7	0.06	0.96	0.31	0.32	0.20	2.65	0.49	0
		Person in charge	3	0.62	2.53	1.29	1.08	1.04	2.16	7.09	0
		Rock breaker	5	0.14	3.22	1.10	1.29	0.59	3.56	2.89	20
		Shift boss	3	0.16	0.53	0.39	0.20	0.35	1.96	1.87	0
	Ore tip	Person in charge	2	0.09	0.20	0.15	0.78	0.13	1.76	2.14	0
		Rock breaker	14	0.05	3.39	1.10	0.96	0.65	3.45	1.34	7
		Section boss	2	0.28	0.69	0.45	0.29	0.40	1.89	1.35	0
		Shift boss	1	1.06	1.06	1.06	0.00	1.06	1.00	0	0
	Loading box	Skip man	9	0.13	3.03	0.62	0.91	0.39	2.33	0.75	11
	Primary crusher	Operator	5	0.12	2.29	0.64	0.94	0.30	3.60	1.49	0
Concentrator	Tertiary crusher	Attendant	11	0.78	1.61	1.51	0.42	1.10	1.44	2.72	0
		Shift boss	2	0.91	5.07	2.99	2.94	2.15	3.37	1,178	50
		Workman	2	0.47	1.66	1.07	0.84	0.88	2.20	2.04	0
		operator	4	0.47	1.67	0.98	0.49	0.89	1.68	0	0
	Dams	Attendant	2	0.17	0.52	0.35	0.25	0.29	2.20	361	0
		Operator	2	0.41	0.73	0.57	0.23	0.55	1.50	21.4	0
	Flotation	Attendant	2	0.26	0.49	0.38	0.16	0.36	1.57	20.0	0
		Operator	3	0.11	1.78	0.79	0.71	0.53	3.19	3.36	0
		Workman	1	0.09	0.09	0.09	0.00	0.09	1.00	0	0
	Foreign ore bin	Attendant	3	1.92	1.92	1.92	0.00	1.92	1.00	0	0
		Operator	4	0.11	1.78	0.79	0.71	0.53	3.19	3.36	0
		Shunter	2	4.12	4.43	4.28	0.22	4.27	1.05	6.77	100
	Screening	Attendant	1	0.37	0.37	0.37	0.00	0.37	1.00	0	0
		Loader driver	1	0.30	0.30	0.30	0.00	0.30	1.00	0	0
		Operator	1	0.27	0.27	0.27	0.00	0.27	1.00	0	0
		Shift boss	1	1.04	1.04	1.04	0.00	1.04	1.00	0	0
		Workman	3	0.90	10.8	6.10	4.94	4.10	3.55	95.2	67
	Secondary crusher	Attendant	2	0.56	5.70	3.13	3.63	1.79	5.16	4,508	50
		Operator	5	0.66	14.0	3.76	5.75	1.81	3.42	8.31	20
		Workman	2	0.22	1.32	0.77	0.78	0.54	3.55	4,233	0
Mining Shaft B	Ore tip	Loco driver	8	0.06	1.13	0.57	0.42	0.39	2.89	0.94	0
		Person in charge	8	0.15	3.09	0.33	0.16	0.29	1.81	1.28	13
		Rock breaker	6	0.04	2.04	0.85	0.75	0.49	4.08	2.15	0
		Section boss	2	0.15	0.42	0.29	0.19	0.25	2.07	174	0
		Shift boss	2	0.46	1.66	1.06	0.85	0.87	2.48	3,036	0
		Whistle man	4	0.49	1.79	0.88	0.61	0.76	1.80	1.93	0
		Workman	15	0.10	2.79	0.62	0.79	0.36	1.80	1.93	0
	Loading box	Workman	5	0.20	0.38	0.29	0.07	0.28	2.77	0.66	0
	Tramming	Loader driver	2	0.74	0.76	0.75	0.01	0.75	1.02	0.89	0
	Sump and waste bin	Loco driver	4	0.11	1.51	0.62	0.62	0.41	2.98	2.33	0
		Person in charge	10	0.19	1.86	0.46	0.51	0.34	2.03	0.20	0
		Rock breaker	7	0.04	2.53	0.71	0.81	0.33	4.57	1.33	0
		Whistle man	3	0.03	2.26	0.78	1.28	0.14	11.2	56.4	0
		Workman	15	0.03	8.05	0.87	2.00	0.31	3.74	0.64	7
	Pump and filter chamber	Operator	6	0.06	0.59	0.25	0.19	0.19	0.08	0.46	0
		Workman	7	0.12	0.67	0.34	0.17	0.30	0.19	0.49	0

Concentrations, time-weighted averages (TWA) of respirable dust exposure; *n*, number of measurements; Min, minimum; Max, maximum; AM, arithmetic mean; SD, standard deviation; GM, geometric mean; GSD, geometric standard deviation; 95% UCI, 95% upper confidence limit of the GM; *n* > OEL, number of measurements above the occupational exposure limit.

**Figure 1:**
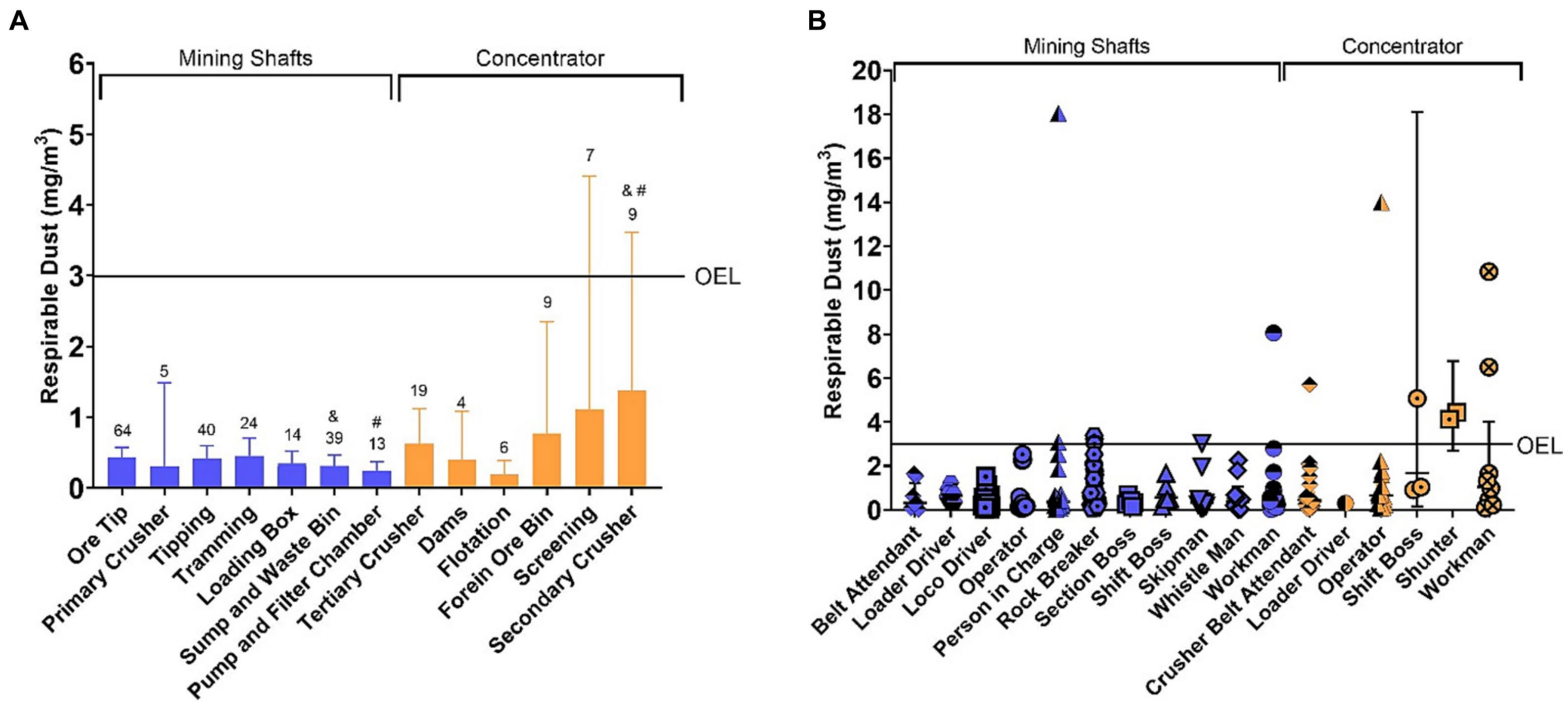
Personal respirable dust exposure in panel **(A)** work areas and panel **(B)** job categories at the mining shafts and concentrator plant. OEL refers to the time-weighted average - occupational exposure limit of 3 mg/m^3^. **(A)**: Numbers indicate the number of samples collected in each work area, and the symbols indicate significant statistical differences between areas. The columns indicate the GM, and the bars indicate a 95% confidence interval. **(B)**: The shapes indicate concentrations from individual measurements, while the horizontal lines indicate the GM, and the bars have a 95% confidence interval.

A one-way ANOVA was used to compare the results from work areas and job categories at the mining shafts and concentrator. The results showed no significant differences in most work areas except between the secondary crusher at the concentrator plant, the sump and waste bin, and the pump and filter chamber at the mining shafts (see Figure 1A). Similarly, job categories were compared to determine if there were significant differences in personal respirable dust exposure among jobs in the mining shafts and at the concentrator (see Figure 1B). After analysis, it was found that there were no significant differences in terms of personal respirable dust exposure among the different job categories. Figure 1 summarizes personal respirable dust exposure in (a) work areas and (b) job categories at the mining shafts and concentrator plant.

When exposure concentrations were then stratified into years. It was observed that the work areas that were sampled in the consecutive years were not the same, and the number of samples collected in each year was not consistent throughout the studies period (this has been summarized in Table 1).

## 4 Discussion

This is the first study to analyze personal respirable dust exposure data collected from a Zambian copper mine and aims to highlight jobs and work areas where workers are at risk of high exposure to respirable dust. Additionally, the study aimed to evaluate the mine’s monitoring program and highlight areas for improvement.

It is important to note that respirable dust exposure sampling at the mining site were not performed according to a standardized sampling protocol. According to EN689, which deals with the measurement of exposure to chemical agents in the workplace, the sampling strategy followed should be fit for purpose in order to test compliance with the exposure limits (22). In the discussion below it will be highlighted that, although the sampling protocol was flawed, the data collected at the mine can be used to identify and prioritize certain work areas and job titles that showed higher exposure compared to other areas. This prioritization can then form part of the basic characterization of exposure risk which is required by standardized protocols like EN689 (22). Since the Zambian mining sector is in its infancy with regards to exposure monitoring and still developing its occupational hygiene capacity, the available data should be utilized to its fullest capacity while understanding the shortcomings thereof.

### 4.1 Work areas

The personal respirable dust exposure concentrations reported in this study were collected from 16 work areas in the two mining shafts (A and B) and the concentrator plant of the Zambian copper mine. Even though the GM for all areas were below the TWA-OEL of 3 mg/m^3^, the screening and secondary crusher at the concentrator plant recorded a 95% upper confidence interval (95%UCI) of the GM exceeding the TWA-OEL of 3 mg/m^3^ (see Figure 1A). It can be seen from Table 2 that 14 of the 253 measurements exceeded the TWA-OEL. These measurements were collected in a variety of work areas including tipping, ore tip and loading box from shaft A, the tertiary crusher, foreign ore bin, screening, and secondary crusher from the concentrator plant and ore tip and sump and waste bin from shaft B. The increased exposures could be due to the type of work that occurs in these areas this may increase the risk of having occupational respiratory diseases (13–15). For example, the screening area where the crushed ore is screened for waste material such as wires and scrap metal as it passes through the conveyer belts and this crushed ore generates airborne dust as it is transported on the conveyer belts. In the secondary crusher area, ore is further crushed into smaller pieces which also generates airborne dust. Although the number of samples at the screening and the secondary crusher were 7 and 9, respectively, and only 1 sample was collected between 2020 and 2022 for each work area and some work areas did not record any measurements during this time (see Table 1). This created a gap in the dataset, and it shows that there is a need for the mining company to prioritize these high exposure work areas within the respirable dust monitoring program. Furthermore, the company must implement effective dust suppression methods such as water mist spray so that workers are protected from respirable dust exposure.

### 4.2 Job categories

Several operations in the mining industry expose workers to respirable dust, including drilling, crushing, tipping, blasting, loading and unloading of ore. These operations may involve job titles such as operators, locomotive drivers, mining crews and team leaders (24). The job titles upon whom the highest exposure to respirable dust was measured during the study period were the shift boss, shunter and workman at the concentrator plant. This is consistent with the findings of Matoset al. (16), who recorded high personal respirable dust exposure concentrations for operators, locomotive drivers and team leaders in an open pit mine. These jobs must be prioritized when controlling exposure to respirable dust at the mining site because they exceeded the TWA-OEL for some measurements. The company needs to prioritize personal respirable dust exposure monitoring and suppression methods in areas where these workers operate in both the mining shafts and the concentrator plant. Even though some job categories recorded exposure measurements above the TWA-OEL, there were no statistically significant differences with those job categories that did not record over-exposure. This could have been attributed to the number of samples that varied substantially between job categories. Another factor could have been that there was no standardized procedure followed when conducting sampling in relation to the number of samples for each job category which implies that there is need for implementation of monitoring measures that will give maximum attention to such job categories. The highest exposure measurement (18 mg/m^3^) was recorded at the tipping work area in mining shaft A for person in charge. However, meta-data linking workers with high exposure scenarios at the mining site was missing to describe why the exposure was high in some job categories.

### 4.3 Monitoring strategy used at the mining shafts and the concentrator plant

The personal respirable dust exposure data used in this assessment consisted of measurements collected at two mining shafts and the concentrator plant at a Zambian copper mine between 2017 and 2022. It was observed that the year 2020 had the highest number of measurements recorded for mining shaft A, with most of them from the tipping area, while the concentrator plant recorded the highest number of measurements in the year 2019, with the majority of measurements coming from the secondary crusher work area. Shaft B recorded the highest number of measurements in 2020, with the ore tip recording the highest. Secondly, measurements representing work areas were not consistent throughout the reporting years. For example, four work areas were measured in 2017 and two in 2018 at the concentrator plant. In 2022, only the floatation area, which is considered to be a low-risk area for respirable dust, at the concentrator plant was monitored (see Table 1). This shows inconsistencies in the way monitoring was performed at the mine. Therefore, the total number of measurements collected in many work areas was not representative of exposure in the work area for that specific year. This made the analysis of temporal trends difficult and the study subsequently only focused on identifying high-risk areas and jobs instead of conducting yearly comparisons to study how exposure changes over time. Additionally, the dataset did not contain adequate meta-data linking high exposures with possible causes and there was not enough information available to verify that the sampling procedure followed by the mine adhered to the general performance requirements of standards such as EN482 (22). Therefore, the comparisons with the exposure limits could not be performed with full confidence but the data could be used to identify areas that need to be prioritized by the mine.

Currently, Zambia does not have legislation guiding the standard procedure in which exposure measurements should be collected, and this could have been one of the reasons for the low number of samples collected by the mining company. Compared to strategies such as the European Standard for workplace air monitoring and measurement (EN689), the strategy followed by the mine could not succeed in evaluating worker exposure with a high degree of statistical accuracy. The EN689 standard states that a preliminary test consisting of a set of three exposure measurements should be conducted in a work area. If one of the results from the set exceeds 10% of the TWA-OEL then no decision can be made on compliance, and additional exposure measurements (up to a minimum of six measurements) need to be collected to determine compliance statistically. If all the measurements from the set are below the TWA-OEL, then the work area is considered a low exposure area. If any of the preliminary set of measurements exceed the TWA-OEL then the work area is considered a high exposure area and is non-compliant which should lead to the implementation of control measures and reevaluation (22). Therefore, the implementation of standard monitoring programs such as that stipulated by the EN689 standard, where the results of exposure measurement are linked to decision-making systems, is essential to protect workers from respirable dust exposure. The current historical dataset is insufficient because the number of samples for each work area was not representative of the number of workers, and there was no consistency in the areas measured to make accurate conclusions. Additionally, the measurements need to be incorporated into a monitoring program where exposures exceeding the TWA-OEL lead to investigations and the implementation of control measures. Since there is no legislation guiding exposure monitoring in Zambia, other mining houses in Zambia may be in similar situations with regards to their exposure monitoring programs. Therefore, this analysis may contribute to improving exposure monitoring programs throughout the Zambian mining industry.

## 5 Limitations

This study did have some limitations which mostly pertained to the quality of the respirable dust exposure data gathered by the mine. The respirable dust exposure dataset used in this study did not follow a standard monitoring procedure. This limited the statistical analysis that could be conducted on the data obtained. There were also limited exposure measurements for certain work areas during the reported period (2017–2022). Additionally, the mining site used the 10 mm conductive nylon cyclone (Dorr-Oliver Style) as the size selective sampler along with a polystyrene cassette in the sampling train. It must be noted that different types of cyclones do not follow the respirable convention in the same precise manner and the results from various types of cyclones may differ slightly (25). Additionally, the use of polystyrene cassettes has been known to cause wall losses that may vary considerably between samples (26). Nevertheless, the only other published study to date to have reported respirable dust exposure in the Zambian mining industry, Hayumbu et al. (14), used the same method as described in this study and it is the method currently in use in the Zambian mining industry. The results from the current study can, therefore, be used by the Zambian mining industry for comparative studies.

## 6 Recommendations

Moving forward it is recommended that the Zambian mining industry formulate a standardized sampling procedure where the entire industry uses the same sampling method and strategy to measure exposure to respirable dust. This procedure must be based on international best practices such as EN689. A fundamental step to achieving this goal would be to select an internationally recognized sampling strategy such as EN689 which allows the user to make use of a limited number of samples to demonstrate whether workers are likely or unlikely to be exposed to concentrations of hazardous chemicals that exceed the exposure limits. However, if this strategy is followed it should all aspects of the strategy, such as using a sampling method that is fit for purpose and applicable quality criteria, should be met (22).

## 7 Conclusion

The analysis holds immense significance for the improvement of the dust monitoring strategy used at this particular mining site as well as those used at other mining operations across Zambia. The study carefully evaluated and interpreted the data from the two mining shafts and the concentrator to gain insight into the mine’s current method of monitoring exposure to respirable dust and potential areas for improvement. The results of this study not only help optimize current practices but also pave the way for more informed decision-making and strategic planning concerning the work areas and jobs that are over-exposed. With this comprehensive analysis, we can identify inefficiencies and make recommendations to enhance overall productivity while minimizing exposure to respirable dust. This has the potential to enable a sustainable and productive future for the mine while protecting the health of mine workers.

The insufficient number of samples limited statistical analysis, which could not be effectively performed on the dataset, and only limited conclusions could be drawn from analyzing the historical data at the mine. Even though the sampling strategy followed by the mine was flawed, high-risk jobs were identified at the concentrator, which included the shift boss, shunter and workman. There is, therefore, a need to implement control measures in these work areas. The study ultimately recommends that there is a need for a standard procedure to guide Zambian companies on how exposure measurements should be conducted for companies assessing air quality in workplaces. One of the standard procedures that can be adopted is the European Standard for Workplace Air Monitoring and Measurements (EN689) which specifies a strategy to perform representative measurements of respiratory exposure to hazardous chemicals in workplaces (22).

## Data availability statement

The data analyzed in this study is subject to the following licenses/restrictions: ethical considerations and secrecy of the mine. Requests to access these datasets should be directed to sifanumwaba@yahoo.com.

## Ethics statement

The studies involving humans were approved by Copperbelt University ethical research committee. The studies were conducted in accordance with the local legislation and institutional requirements. Written informed consent for participation was not required from the participants or the participants’ legal guardians/next of kin in accordance with the national legislation and institutional requirements.

## Author contributions

MS: Conceptualization, Investigation, Methodology, Writing – original draft, Writing – review & editing. KK: Conceptualization, Methodology, Project administration, Software, Supervision, Writing – original draft. PH: Conceptualization, Investigation, Methodology. Writing – review & editing. LN: Formal analysis, Investigation, Methodology, Writing – original draft. SL: Conceptualization, Investigation, Methodology, Software, Supervision, Validation, Writing – original draft, Writing – review & editing.
