# Supplementary material for: Analysis of respirable dust exposure data collected at a Zambian copper mine between 2017 and 2022
Source: Front Public Health. 2024 Jan 31;11:1288485. doi: 10.3389/fpubh.2023.1288485 (PMC10864506; doi:10.3389/fpubh.2023.1288485)
Supplement: Supplementary file 1 [file Table_1.docx]

Table S1. Job titles per work area

| **Job Title** | **Work area** | **Number of workers sampled** |
| --- | --- | --- |
| Belt attendant | Shaft A | 7 |
|  | Shaft B | 0 |
|  | Concentrator Plant | 21 |
| Loader driver | Shaft A | 8 |
|  | Shaft B | 2 |
|  | Concentrator Plant | 5 |
| Locomotive driver | Shaft A | 8 |
|  | Shaft B | 12 |
|  | Concentrator Plant | 0 |
| Operators | Shaft A | 7 |
|  | Shaft B | 12 |
|  | Concentrator Plant | 15 |
| Person in charge | Shaft A | 9 |
|  | Shaft B | 0 |
|  | Concentrator Plant | 0 |
| Rock breaker | Shaft A | 25 |
|  | Shaft B | 6 |
|  | Concentrator Plant | 0 |
| Shift boss | Shaft A | 2 |
|  | Shaft B | 4 |
|  | Concentrator Plant | 4 |
| Section boss | Shaft A | 4 |
|  | Shaft B | 2 |
|  | Concentrator Plant | 0 |
| Skip man | Shaft A | 14 |
|  | Shaft B | 0 |
|  | Concentrator Plant | 0 |
| Whistle man | Shaft A | 3 |
|  | Shaft B | 7 |
|  | Concentrator Plant | 0 |
| Work man | Shaft A | 0 |
|  | Shaft B | 43 |
|  | Concentrator Plant | 9 |
